# The role of attentional focus on walking efficiency among older fallers and non-fallers

**DOI:** 10.1093/ageing/afz113

**Published:** 2019-10-03

**Authors:** Toby C T Mak, William R Young, Wing-Kai Lam, Andy C Y Tse, Thomson W L Wong

**Affiliations:** 1 School of Public Health, Li Ka Shing Faculty of Medicine, The University of Hong Kong, Hong Kong SAR, China; 2 Institute of Environment, Health and Societies, Brunel University London, UK; 3 Department of Clinical Sciences, Brunel University London, UK; 4 College of Life and Environmental Sciences, University of Exeter, UK; 5 Li Ning Sports Science Research Center, Li Ning (China) Sports Goods Co. Ltd, Beijing, China; 6 Department of Kinesiology, Shenyang Sport University, Shenyang, China; 7 Department of Health and Physical Education, The Education University of Hong Kong, Hong Kong SAR, China

**Keywords:** falls, gait, muscle, efficiency, attention, older people

## Abstract

**Background:**

This study evaluated the effect of attentional focus instructions on movement efficiency during a level-ground walking task in older adults with and without a history of falls.

**Methods:**

One hundred and thirty-four community-dwelling older adults were categorised into older fallers (OF) (*n* = 37) and older non-fallers (ONF) (*n* = 97). Each participant was instructed to walk at a self-selected pace along a 6 m walkway under three attentional focus conditions (i.e. internal, goal-directed and control) for a total of nine trials. Average muscle activity indices of lower limb co-contractions were measured using surface electromyography.

**Results:**

Both shank and thigh muscle co-contractions were higher in OF than in ONF in all three conditions. OF also demonstrated higher shank muscle co-contraction under the internal relative to the goal-directed condition, with no such change observed in ONF.

**Conclusion:**

Despite no significant between-group differences in functional balance and balance confidence, relative walking inefficiencies were observed in OF compared with ONF. This finding demonstrates the debilitating consequences of falling that can occur with relative independence from various physiological or psychological factors that are commonly associated with falling and used to rationalise behavioural change. We also provide evidence that OF are more susceptible to conditions that provoke them to allocate attention internally. Therefore, in clinical contexts (e.g. gait rehabilitation), verbal instructions that refer to body movements (internal focus) might serve to compromise movement efficiency in older adults with a history of falls. Such changes will, theoretically, lessen the ability to react efficiently to changing environments experienced in daily life.

## Key points

- The effect of attentional focus instructions on walking efficiency was evaluated between fallers and non-fallers.
- Lower limb muscle co-contractions differ between fallers and non-fallers.
- Differences in muscle co-contractions appear to be unrelated to balance ability and balance confidence.
- Internal focus instructions compromise walking efficiency in fallers by virtue of a ‘stiffening strategy’.
- A cautious ‘stiffening strategy’ may be associated with reduced gait stability in older fallers

## Introduction

The ability to walk safely and efficiently is essential for older adults to maintain independence and minimise fall risk [1]. However, age-related changes in the neuromuscular system—relating to both muscle activation and strength—compromise movement efficiency, thus contributing to instability during gait [2], leading to increased fall risk [3]. Consequently, there is an urgent need to evaluate the multifactorial factors that influence movement efficiency and walking stability in older adults.

In recent years, research in motor control has examined the influence of altered attentional focus on motor performance, where guiding/instructing performers to adopt an external/goal-directed attentional focus is generally associated with improved performance and efficiency [4]. In contrast, internal focus of attention (towards monitoring and controlling movement) often results in compromised movement success and motor efficiency. For example, Vance and colleagues [5] instructed young participants to carry out biceps curls in external and internal focus conditions. For external focus, participants were asked to pay attention to the biceps curl bar, whereas, for the internal focus condition, they were asked to focus on arm movement. Compared with internal focus, external focus trials led to reduced average electromyography (EMG) activity in biceps brachii with the same weight lifted, indicating greater motor efficiency. These findings have since found support from studies observing neuromuscular efficiency in sporting tasks (e.g. basketball free throwing [6] and vertical jump-and-reach tasks [7]), with all studies promoting the perspective that internal focus leads to ‘noise’ in the motor system and associated performance disruption.

Although these previous studies pointed to the fact that movement efficiency varies significantly with an individual’s focus of attention in younger adults, it remains unclear how such effects of attentional focus relate to movement efficiency in older adults. Assessment of EMG lower limb co-contraction delivers a direct indication of movement efficiency during walking in older adults [8]. Indeed, previous literature suggests that excessive/unnecessary co-activation in older adults is likely to cause higher energy costs/inefficiencies during locomotion, which subsequently induce fatigue and lead to a higher likelihood of falls [9].

Based on previous observations of increased lower limb muscle co-activation patterns during walking in female older fallers [10], we sought to examine whether neuromuscular efficiencies differ between older fallers (OF) and older non-fallers (ONF) in gait. Primarily, we predicted that higher muscle co-contractions will be found in OF compared with ONF. These potential differences could be the consequences of numerous interrelated, physical and psychological factors between groups. In addition, given the clear association between altered attentional focus and changes in both muscle efficiencies and performance outcomes in non-gait tasks, we also aimed to investigate the effect of attentional focus instructions on movement efficiency of level-ground walking in older adults. Based on previous observations that OF show a propensity to consciously control movement [11], we predicted that OF would be more susceptible to instructions to focus internally, which, in turn, will lead to relative increases in muscle co-contractions.

## Methods

### Participants

One hundred and thirty-four community-dwelling older adults (94 females, 40 males) (mean age = 70.3 ± 4.8) participated in the study (Table 1). They were recruited by convenience sampling from community centres in Hong Kong. Participants with any history of neurological impairment were excluded. Participants were also excluded if they scored less than 24/30 on the Chinese version of the Mini-Mental State Examination (MMSE-C) [12], or acquired static visual acuity worse than 20/40 vision. All participants were able to walk independently indoors. Participants with any history of falls that resulted in unintentionally landing on the ground within the past year were categorised as OF (*n* = 37; 7 males, 30 females) [13]. Participants without any previous incidence of falling were categorised as ONF (*n* = 97; 33 males, 64 females). Fall history was defined as any fall incident which can be clearly recalled in terms of time frame, location and mechanism. Prior to participation, all participants provided their written informed consent.

**Table 1:** Participants’ characteristics (*n* = 134).

Variables	Mean (SD) or *n* (%)	
	Older fallers (OF)	Older non-fallers (ONF)
*n* (numbers)	37	97
Gender (female)	30 (81.1%)	64 (66.0%)
Age (years)	70.7 (5.0)	70.1 (4.7)
MMSE-C	29.0 (1.4)	29.1 (1.2)
BBS	54.3 (1.6)	54.9 (1.4)
TUG (s)	11.2 (1.9)	10.9 (2.3)
FES-13	113 (12)	118 (13)

*Note*: MMSE-C = Mini-Mental State Examination (Chinese version) (range: 0–30); BBS = Berg Balance Scale (range: 0–56); TUG = Timed Up & Go Test; FES-13 = Falls efficacy scale (13 items) (range: 0–130).

### Apparatus and task

Participants were required to walk along a 6 m level-ground walkway at their natural pace under three attentional focus conditions (internal, goal-directed and control). A 27˶ LED computer-linked monitor was situated shortly after the end of the walkway and was referred to as the destination of each walking trial (available for goal-directed condition).

Muscle activity was measured by surface EMG with a wireless telemetric system (TeleMyo DTS, Noraxon Inc., Scottsdale, AZ, USA) at 3 kHz. Pre-gelled bi-polar Ag/AgCl circular electrodes (Noraxon Dual Electrodes, Noraxon, AZ, USA) (diameter, 10 mm; interelectrode spacing, 17.5 mm) were attached over four muscles on each leg: tibialis anterior (TA), medial gastrocnemius (MG), biceps femoris (BF) and rectus femoris (RF), following the SENIAM guidelines [14]. Each sensor electrode had a differential amplifier attached (specification: input range ±3.5 mV, input impedance >100 MX, common mode rejection >100 dB, base gain of 500 and baseline noise <1 Lv root mean square). The analogue signal was hardware bandpass filtered (10–500 Hz) and converted to digital signal by the transmitter data acquisition system (16 bit).

### Procedure

Clinical baseline measurements were first collected (Table 1). Functional mobility was evaluated by the Timed Up & Go (TUG) test, where a time of ≥14 s to complete the task indicates a high fall risk [15]. The Berg Balance Scale (BBS) assessed functional balance [16]. Higher scores represent better balance ability. Falls efficacy was evaluated using the Falls Efficacy Scale (FES-13 items) [17]. A higher score represents a higher degree of self-confidence to participate in normal daily activities without falling.

Before the start of the walking trials, maximal voluntary isometric contractions (MVICs) of targeted lower limb muscles (TA, MG, BF and RF) were recorded by EMG according to the protocol described by Hsu and colleagues [18] for normalisation of EMG signals acquired during the walking task. Participants then carried out three practice trials before completing a total of nine walking trials. The nine trials comprised three repetitions of the three different attentional focus instructions. The order of attentional focus conditions was randomised across participants. For the control condition, no other specific instruction was given. For the internal condition, participants were instructed to ‘Please focus on your lower limb movement when walking’. For goal-directed condition, we adopted an instruction based on concepts described by Wulf and colleagues’ [19], with the main purpose of encouraging a goal-related focus on movement effects. Participants were instructed to ‘Please focus on the random series of digits ranging from 1 to 9 that will be presented on the computer monitor at your destination when walking’ [20]. The monitor was only switched on under goal-directed trials.

### Data analysis

EMG data were processed using MyoResearch 3.8.6 (Noraxon Inc., Scottsdale, AZ, USA). Data collected during MVIC and walking trials were filtered using a 20–500 Hz bandpass filter, full-wave rectified and smoothed with the root mean square (RMS) algorithm with a 100 ms window. The peak value for MVIC was averaged over a 500 ms window, and was used to normalise the amplitude of EMG signals of the respective muscles from the walking trials. Normalised EMG signals were used to determine the co-contraction index (CCI) of lower limbs; a dimensionless value that allowed comparison of co-contraction levels among individuals and groups [21]. The co-contraction indices were calculated using the overlapping area of normalised EMG signals of MG and TA (for shank), and BF and RF (for thigh), divided by the number of data points [21]. All calculations were completed using customised Matlab program (R2015b, Mathworks Inc., USA).

Statistical analysis was performed using SPSS version 23.0. One-way analysis of covariances (ANCOVAs) were performed to compare co-contractions (CCI) of different muscle groups (i.e. thigh and shank) under the control condition between OF and ONF after adjusting for covariates of cognitive functioning, functional mobility, functional balance and balance confidence. Subsequently, two-way RM-ANOVAs with Bonferroni adjusted post-hoc tests were used to assess the effects of group (OF and ONF) and condition (control, internal and goal-directed) on the co-contractions (CCI) of different muscle groups (i.e. thigh and shank).

## Results

### Faller versus non-fallers

Results showed significant differences in co-contractions between OF and ONF for both shank and thigh muscle groups after adjusting for all the covariates (*F* [5, 128] = 2.505, *P* = 0.034; *F* [5, 128] = 2.682, *P* = 0.024). All covariates were not significant in both muscle group comparisons (all *P* > 0.05). OF demonstrated significantly higher lower limb muscle co-contractions than ONF.

### Co-contraction of shank muscle groups

**Figure 1:**
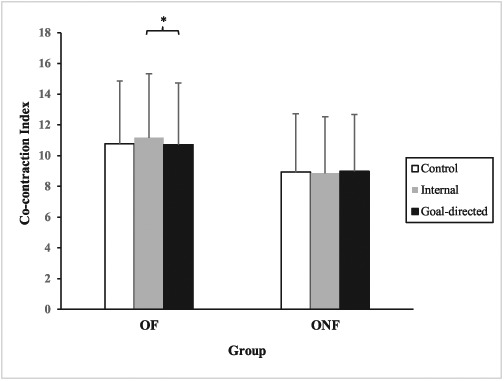
Co-contraction index of shank for older faller (OF) and older non-faller (ONF) groups under control, internal and goal-directed conditions. ^*^ Denotes significant difference (*P* < 0.05).

Results showed a significant main effect of group, whereby OF demonstrated a significantly higher level of co-contractions across all conditions, compared with ONF (*F* [2, 264] = 6.933, *P* = 0.010). There was no significant main effect of condition. However, there was a significant two-way interaction between group and condition (*F* [1.909, 244.371] = 3.615, *P* = 0.028). Post-hoc comparisons revealed that, while no significant differences were observed between conditions in ONF, OF demonstrated significantly greater co-contractions during internal compared with goal-directed condition (Figure 1).

### Co-contraction of thigh muscle groups

**Figure 2:**
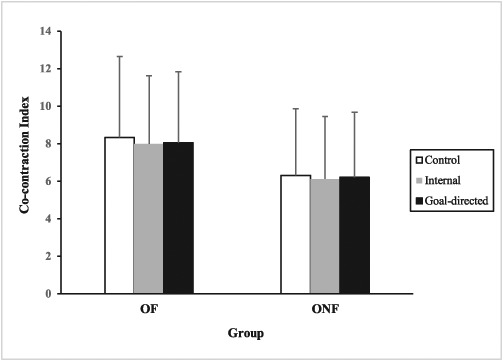
Co-contraction index of thigh for older faller (OF) and older non-faller (ONF) groups under control, internal and goal-directed conditions.

The results showed main effects of both group (*F* [2, 256] = 10.882, *P* = 0.001) and condition (*F* [2, 256] = 4.106, *P* = 0.018). Post-hoc analyses revealed that, across all three conditions, co-contractions were greater in OF compared with ONF. Surprisingly, across both groups, co-contractions were greater in the control relative to the internal condition (*P* = 0.027) (Figure 2).

## Discussion

The present study evaluated differences in neuromuscular efficiencies, as indicated by lower limb muscle co-contractions, between OF and ONF during level-ground walking. Our data show that lower limb muscle co-contractions were higher in OF compared with ONF after largely eliminating the effect of functional mobility, balance ability and balance confidence. As such, differences observed in muscle co-contractions appear to be primarily due to fall history (and/or other potential unmeasured factors) rather than physiological and psychological changes that are commonly assumed to mediate differences in gait patterns and efficiencies. After experiencing a fall, we might intuitively expect older adults to adopt a more ‘cautious’ approach during posture and gait tasks in an attempt to protect themselves from future falls [22]. Clear evidence currently points to an association between fall history and cautious gait (typically characterised by slower speed, shorter steps and increased joint stiffness) [3,22], an association presumably underpinned, at least in part, by co-contraction-related increases in joint stiffness [23]. The magnitude of differences observed in muscle co-contractions might appear to be relatively small. However, previous research by Lo and colleagues [24] demonstrated that even seemingly trivial increases in co-contraction are significantly associated with increased swing and stance durations. The clinical importance of increased co-contraction not only relates to consequential alterations in gait characteristics, but also to increased risk of falls by increasing lower limb rigidity/stiffness which impedes reactive adaptation to postural perturbations [22,25]. We cannot provide specific thresholds on the CCI for each antagonist muscle group that might represent a clinically meaningful change. While existing literature suggests that even a small change (<5% change in co-contractions observed in Lo *et al*. [24]) might be necessary to induce significant behavioural changes and influence movement efficiency, future work should endeavour to identify such thresholds so that clinicians might avail of such measures when evaluating neuromuscular efficiency in patients.

Clinicians typically use a battery of tests that quantify physiological, functional and cognitive factors thought to influence the risk of future falls [26]. These clinical tests, such as TUG, are presumed to discriminate between individuals who have low/high risk for falls [15]. However, all our participants, even those with a history of falls, are categorised as being at a low risk of falling based on their relatively high functional ability. While a history of falls is clearly associated with an individual’s future fall risk [27], we argue that these clinical tests may have limited ability to identify relatively high fall risk individuals who have strong cognitive and physical functioning. Future studies should focus on collecting prospective fall data to examine the predictive value of measuring neuromuscular efficiency, not only to evaluate the potential contribution to improving predictions of future falls, but also to establish more insightful methods for monitoring recovery in rehabilitation following a fall, especially in older adults with pronounced fear of falling.

In the current study, OF appeared to be more vulnerable to the effect of internal focus than ONF, exhibiting increased shank muscle co-contraction under internal relative to goal-directed conditions. The increased susceptibility to internal focus instruction shown in OF appears to be largely independent of general physical ability (see Table 1). Faes and colleagues [28] investigated ‘post-fall’ impact in older persons using qualitative methods and described OF as having higher propensity to reflect on the attribution of their falls, assigning ‘blame’ to their increased age and intrinsic factors, such as physical condition. This served to enhance their awareness of their own limitations, regardless of their actual physical state. We speculate that ruminations about previous falls could, independently from balance confidence (based on between-group similarities in FES-13 scores), lead to the development of new movement ‘rules’ (e.g. ‘pick your feet up, keep your back straight’), designed to guard against the perceived physiological ‘culprit’ for the previous fall. Following the development of such verbal cues, we suggest that OF were better equipped to adopt the experimental internal focus instructions, resulting in a more cautious but less efficient gait. Interestingly, greater co-contractions were only observed in shank but not thigh muscle groups under the internal condition in OF. It appears that, even during a relatively simple walking task, OF prioritise maintaining ankle joint stability by co-contracting ankle-stabilising muscles over maintaining knee joint stiffness under the internal condition.

There were limitations to our study. While it was advantageous to compare OF and ONF groups in the relative absence of physical and cognitive differences, the relatively high functioning of both groups creates difficulties in generalising current findings to older adults with pronounced muscle weakness or reduced balance confidence. We did not measure kinematic data concerning joint range of motion in the lower limbs. Such information could enhance our understanding relating to joint stiffness and muscle tension of the ankle engagement. Additionally, our goal-directed focus instructions did not induce any improvement of neuromuscular efficiency from the control condition. We suggest further study to include alternative distraction manipulations, such as emotional expression (e.g. smiling), for encouraging relaxation which potentially reduces co-contractions in the lower limbs [29].

In conclusion, our results provide novel information about factors influencing fall-related differences in movement inefficiency. The results demonstrate clear differences in muscle co-contractions between OF and ONF in the relative absence of disparities between factors commonly associated with fall risk. We also reinforce the view that instructions which induce internal focus potentially compromise movement efficiency by virtue of a ‘stiffening strategy’ adopted by OF. We argue that such changes may be associated with reduced gait stability in OF [30], and that such a strategy would represent an increased threat during more demanding tasks (i.e. where dynamic and/or rapid responses are required). From a clinical perspective, we suggest that instructions referring to body movements could potentially be detrimental to patients’ movement efficiency and stability. Further work is necessary to establish functional consequences of such inefficiencies.

### Ethics

The study was approved by the Human Research Ethics Committee for Non-Clinical Faculties at the University of Hong Kong (Reference Number: EA1501054).
